# Neural Mechanisms Underlying Perilesional Transcranial Direct Current Stimulation in Aphasia: A Feasibility Study

**DOI:** 10.3389/fnhum.2015.00550

**Published:** 2015-10-07

**Authors:** Lena Ulm, Katie McMahon, David Copland, Greig I. de Zubicaray, Marcus Meinzer

**Affiliations:** ^1^Centre for Clinical Research, The University of Queensland, Brisbane, QLD, Australia; ^2^Centre for Advanced Imaging, The University of Queensland, Brisbane, QLD, Australia; ^3^School of Health and Rehabilitation Sciences, The University of Queensland, Brisbane, QLD, Australia; ^4^Faculty of Health, Institute of Health and Biomedical Innovation, Queensland University of Technology, Brisbane, QLD, Australia

**Keywords:** aphasia, stroke, anomia, transcranial direct current stimulation, functional magnetic resonance imaging

## Abstract

Little is known about the neural mechanisms by which transcranial direct current stimulation (tDCS) impacts on language processing in post-stroke aphasia. This was addressed in a proof-of-principle study that explored the effects of tDCS application in aphasia during simultaneous functional magnetic resonance imaging (fMRI). We employed a single subject, cross-over, sham-tDCS controlled design, and the stimulation was administered to an individualized perilesional stimulation site that was identified by a baseline fMRI scan and a picture naming task. Peak activity during the baseline scan was located in the spared left inferior frontal gyrus and this area was stimulated during a subsequent cross-over phase. tDCS was successfully administered to the target region and anodal- vs. sham-tDCS resulted in selectively increased activity at the stimulation site. Our results thus demonstrate that it is feasible to precisely target an individualized stimulation site in aphasia patients during simultaneous fMRI, which allows assessing the neural mechanisms underlying tDCS application. The functional imaging results of this case report highlight one possible mechanism that may have contributed to beneficial behavioral stimulation effects in previous clinical tDCS trials in aphasia. In the future, this approach will allow identifying distinct patterns of stimulation effects on neural processing in larger cohorts of patients. This may ultimately yield information about the variability of tDCS effects on brain functions in aphasia.

## Introduction

Chronic language impairments (aphasia) are among the most devastating consequences of stroke. Given that even specific- and deficit-oriented therapy delivered with high intensity may only result in moderate treatment effect sizes (Kelly et al., 2010), there is a need to explore new strategies to enhance treatment efficacy. Transcranial direct current stimulation (tDCS) may be a promising tool to achieve this goal (De Aguiar et al., 2015). During tDCS, a weak electrical current is projected between two scalp affixed electrodes which modulates cortical excitability (Stagg and Nitsche, 2011). tDCS is a simple to use and low cost technology with an excellent safety profile. In healthy individuals, it has been demonstrated that excitatory anodal-tDCS administered to left perisylvian brain areas can improve language processing (Iyer et al., 2005; Floel et al., 2008; Sparing et al., 2008; Meinzer et al., 2012a, 2013) when compared to placebo stimulation (sham-tDCS). Repeated stimulation sessions may result in long-lasting enhancement of motor or cognitive learning (Reis et al., 2009; Cohen Kadosh et al., 2010). Thus, anodal-tDCS may also be suited to enhance the recovery potential in aphasia.

A growing number of studies have combined anodal-tDCS with speech therapy in aphasia and one of the most promising approaches may involve the stimulation of perilesional brain regions that have been shown to be important for spontaneous and treatment-induced recovery (Meinzer et al., 2008; Fridriksson, 2010). In two sham-tDCS controlled trials, anodal-tDCS was administered to perilesional brain regions that were identified by pre-treatment functional magnetic resonance imaging [fMRI (Baker et al., 2010; Fridriksson et al., 2011)] and anodal-tDCS enhanced the outcome of a naming treatment in chronic patients with aphasia compared to treatment with sham-tDCS. However, the neural mechanisms underlying those behavioral effects were not assessed and tDCS effects were variable, with some individuals not showing benefits. Thus, a better understanding of how tDCS impacts language functions in aphasia is necessary to optimize future stimulation trials.

Recent technical advances allow this issue to be addressed by administering tDCS during simultaneous fMRI to elucidate the neural underpinnings of acute stimulation effects (Meinzer et al., 2014a). A number of studies have successfully used this technique to study the neural underpinnings of language improvement due to anodal-tDCS in healthy individuals (Holland et al., 2011; Meinzer et al., 2012a, 2013, 2014b). In the present proof-of-principle study, we used this novel method to explore the feasibility to target an individualized stimulation site during intrascanner tDCS to assess the neural mechanisms underlying perilesional anodal-tDCS in an individual with post-stroke aphasia.

## Materials and Methods

### Study overview

**Figure 1:**
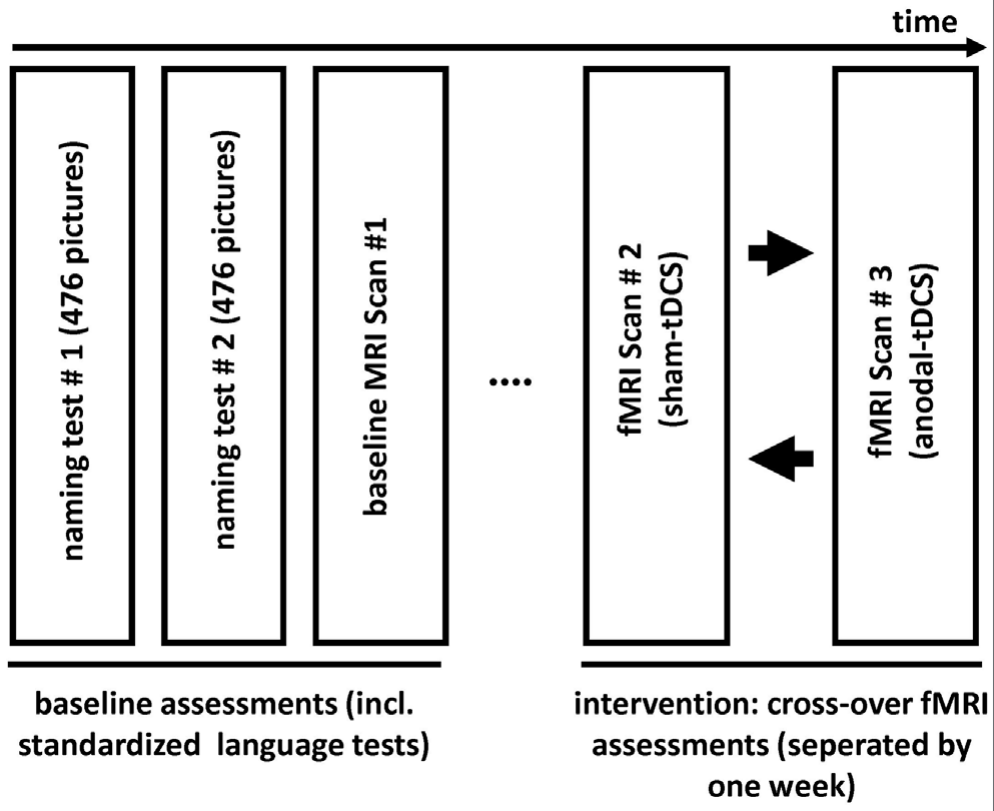
**Illustrates the design of the study**.

This single-subject, sham-tDCS controlled study employed an overt picture naming task because impaired word-retrieval (anomia) is the most frequent symptom in chronic aphasia and frequently targeted in therapy (Kelly et al., 2010; Klebic et al., 2011). As in previous clinical trials (Baker et al., 2010; Fridriksson et al., 2011), the stimulation targeted spared perilesional regions that were identified during a baseline fMRI scan. Figure 1 illustrates the study design. Initially, the degree of language impairment was determined using standardized language tests and the patient participated in two separate behavioral baseline testing sessions during which a large picture naming battery was administered twice. Based on the results of the baseline naming sessions, three sets of 80 pictures that the patient could name spontaneously were selected and matched for linguistic criteria and naming latency during the baseline sessions (see below). The three sets were presented during three subsequent fMRI sessions: A *baseline fMRI session* aimed to determine brain activity patterns associated with correct naming attempts during an overt picture naming task. During a subsequent *cross-over phase*, the remaining two picture sets were named by the patient during the same fMRI-task either with simultaneous anodal-tDCS or sham-tDCS. The cross-over scans were scheduled 1 week apart. During both stimulation sessions, the active electrode (i.e., anode) was centered over peak activity elicited by correct naming trials during the baseline fMRI session. The comparison of the two stimulation conditions assessed potential effects of tDCS on functional brain activity.

For this proof-of-principle study, we recruited a patient with mild aphasia and only used pictures that could be named correctly during the baseline assessments, thereby accounting for day-to-day variability in naming performance to assure stable imaging results. In order to be able to attribute potential activity differences to anodal-tDCS, we also aimed to minimize the impact of performance differences between sessions by closely matching the respective sets of pictures, as performance levels impact on the degree of functional activity during language tasks (Meinzer et al., 2012c). Importantly, our set-up does not address the impact of the stimulation on performance, however, it reveals which brain regions are affected by a given montage on an individual basis to identify regions, which may potentially interact with speech therapy effects. Such information would allow targeted assignment of patients to specific therapies designed to engage specific neural circuits that are rendered “more responsive” by the stimulation.

### Patient characteristics

**Figure 2:**
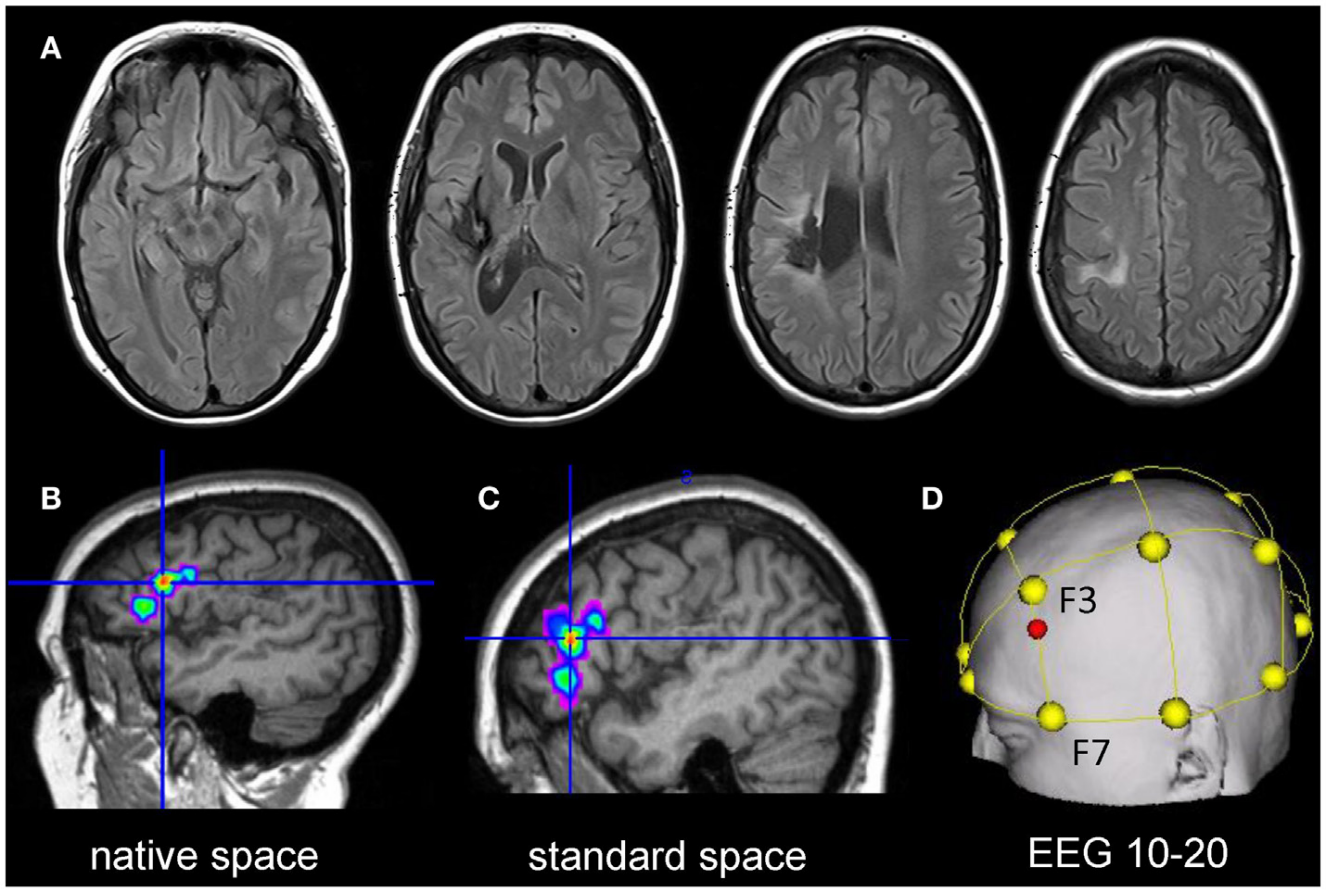
Upper panel: **(A)** shows the structural lesion of the patient in native space (left = left). Lower panel: illustrates the procedure to determine the individual stimulation site for the patient: **(B)** the peak cluster obtained during the baseline fMRI session is shown overlaid on the patient’s co-registered T1-weighted image in native space using MRIcron (http://www.mccauslandcenter.sc.edu/mricro/mricron/). **(C)** Subsequently, the peak cluster is converted into standard space using the normalization parameters generated during segmentation of the structural T1-weighted scan. **(D)** Normalized peak coordinates are transferred into EEG 10–20 system coordinates using the Münster T2T converter (red dot illustrates location of the center of the electrode on the scalp).

A 51-year-old, right-handed female native English speaker with mild chronic aphasia (4.7 years post-stroke) following a left-sided ischemic stroke was recruited. Structural imaging revealed a left-sided lesion affecting the posterior insula, superior temporal and inferior parietal lobe, and the neighboring white matter (Figure 2A). Standardized testing revealed mild naming impairment on the Boston Naming Test [(Kaplan et al., 1983) 52/60 correct responses] and the patient had mild anomic aphasia (Kertesz, 2006). The study was approved by the ethics committee of The University of Queensland and was conducted in accordance with the Helsinki declaration. The patient provided written informed consent prior to study inclusion.

### Baseline naming assessments

During two baseline naming assessments, 476 object pictures (black and white line drawings) from the International Picture Naming Project Database (Szekely et al., 2004) were presented in random order using a laptop computer. Pictures were presented in 10 sets of approximately 48 pictures with breaks in between sets. Each picture was preceded by a brief auditory stimulus and presented for up to 8 s. The patient was asked to name each picture aloud during this time as quickly and accurately as possible and she could proceed at her own speed after each naming attempt. Verbal responses were recorded and transcribed. Response latencies for correctly named pictures were determined using Audacity© software (by calculating the difference between picture onset, identified by the auditory stimulus, and the onset of correct naming responses).

### Stimulus selection

Of pictures that could be named correctly during both baseline assessments (*N* = 388/476), the 240 responses with the fastest mean latency were selected and divided into three sets (80/set, matched for baseline response latency mean ± SD seconds Set_1_: 2.49 ± 0.32 Set_2_: 2.53 ± 0.37 Set_3_: 2.59 ± 0.57; *p* = 0.37) and linguistic variables (number of alternative names and semantic categories, name agreement/mean reaction time norm group, length, frequency, age of acquisition; *p* = 0.44 to 0.93). Sets were randomly assigned to one of the fMRI sessions (baseline = Set_3_, anodal-tDCS = Set_2_, sham-tDCS = Set_1_).

### Functional imaging data acquisition

Functional and structural images were acquired using a 3-T Siemens Trio MRI system with 1 week between scanning sessions. The overt picture naming task employed a T2*-weighted echo-planar imaging sequence (TR/TA: 12,000/2500 ms, 9500 delay of TA; echo time 36 ms, matrix 64 × 64, 36 mm × 3mm slices, 0.3 gap, flip angle 80, in-plane resolution 3.28 mm × 3.28 mm × 3.3 mm, 2 × 61 volumes) with a sparse acquisition design (window length/order = 1, Gaab et al., 2007). This allows assessing overt verbal responses during a scanner off phase to avoid articulation related artifacts. Picture stimuli were presented using Matlab^®^ and Cogent software using a projector and a system of mirrors. During each trial, a blank screen was displayed for 4500 ms, followed by a fixation cross (500 ms) and an object picture (3000 ms). The patient was instructed to name each picture aloud as fast as possible during this time. A whole brain volume was acquired 1500 ms after picture offset (inter-stimulus interval 12 s). During each run, pictures were presented in four blocks of 10 consecutive trials. Task blocks alternated with four baseline blocks (scrambled object pictures matched for visual complexity to the object pictures; five consecutive trials). During baseline trials, the patient was instructed to articulate a standard response (“pass”). Overt responses were recorded using an MRI-compatible microphone for subsequent analysis (accuracy, response latency of correct responses). High-resolution 3D T1-weighted and fluid attenuation inversion recovery images were acquired for lesion identification and to facilitate normalization of the functional images (Meinzer et al., 2012b). A training session was conducted outside of the scanner using a different set of pictures.

### Identification of peak activity during the baseline fMRI scan

Statistical Parametric Mapping (SPM8, Wellcome Department of Imaging Neuroscience, London, UK) was used for data analysis. Pre-processing of the data comprised re-alignment, co-registration with the structural image, and spatial smoothing (6 mm × 6 mm × 6 mm Gaussian kernel). Data were initially analyzed in native space. This allowed inspection of the spatial correspondence of activity with regard to local anatomy in comparison with the subsequently normalized images (Meinzer et al., 2012b). Covariates-of-interest included in the statistical design matrix were correct picture naming and baseline trials. Movement parameters were also included to improve the overall model fit. Afterwards, a high-pass filter (128 s) was applied, the data were modeled with a finite impulse response and the contrast-of-interest was estimated (picture naming vs. baseline trials). The resulting statistical map was thresholded at *p* < 0.001 (voxel level) and a family wise-error corrected cluster level of *p* < 0.05. The resulting peak cluster is illustrated in native space in Figure 2B and was located in the left inferior frontal gyrus (IFG). Subsequently, the high-resolution T1-weighted image was warped into standard space using unified segmentation and cost-function masking (Meinzer et al., 2012b). The resulting normalization parameters were applied to the peak cluster (normalized peak activity: *x*/*y*/*z* Talairach coordinates −43/32/11; see Figure 2C). To obtain the location of scalp coordinates for centering the tDCS-electrode over this area, an online tool was used that allows transferring coordinates in standard space into EEG 10–20 system scalp coordinates (http://wwwneuro03.uni-muenster.de/ger/t2tconv/). The resulting target location for placing the electrode corresponded to ~75% upwards from F7 to F3 (Figure 2D).

### Transcranial direct current stimulation

We employed the same stimulation parameters that yielded positive results in previous intrascanner tDCS studies of word retrieval (Meinzer et al., 2012a, 2013, 2015). tDCS was administered with a constant direct current (1 mA) using an MRI-compatible stimulator (DC-Stimulator Plus^®^, NeuroConn) using an established set-up during fMRI (Meinzer et al., 2014a). The anode (5 cm × 7 cm) was attached over the location of peak activity identified during the baseline scan. The cathode (10 cm × 10 cm) was positioned over the right supraorbital region as in previous studies that used a similar intrascanner set-up. By choosing a large (“functionally inert”) reference electrode, we also aimed to avoid complications with interpreting potential effects that would have been associated with a smaller “active” reference electrode (i.e., any effects may be associated with either the active anode, the active cathode or a combination of both). The current was ramped-up over 10 s prior to the start of the picture naming task and remained stable for 20 min (anodal-tDCS, cross-over phase scan 2) or was turned off after 30 s (sham-tDCS, cross-over phase scan 1).

### Functional MRI data analysis cross-over phase

Functional images acquired during both imaging sessions (*S*_2_ = sham-tDCS; *S*_3_ = anodal-tDCS) were spatially re-aligned to the first image of the time series and co-registered. The resulting images were then warped into standard space and smoothed with a 6 mm × 6 mm × 6 mm Gaussian Kernel. The statistical design parameters were identical as for the baseline analysis but included both cross-over imaging sessions to account for different noise levels across sessions (Meinzer et al., 2012b). After high-pass filter, data were modeled with a finite impulse response and the contrasts of interest were estimated (correct naming trials vs. baseline trials for each session and the direct comparison of the two sessions; anodal-tDCS vs. sham-tDCS and vice versa). The resulting contrast images were thresholded at a voxel threshold of *p* < 0.001 (cluster threshold *p* < 0.05, inclusively masked with a binary mask based on the combined significant activity patterns elicited by both scanning sessions; both masks were saved individually and then combined using SPM-ImCalc; voxel threshold *p* < 0.001; cluster level *p* < 0.05).

## Results

The patient reported only minor adverse effects (mild tingling, itching) and could not reliably distinguish between the stimulation conditions.

### Electrode positioning during anodal-tDCS

**Figure 3:**
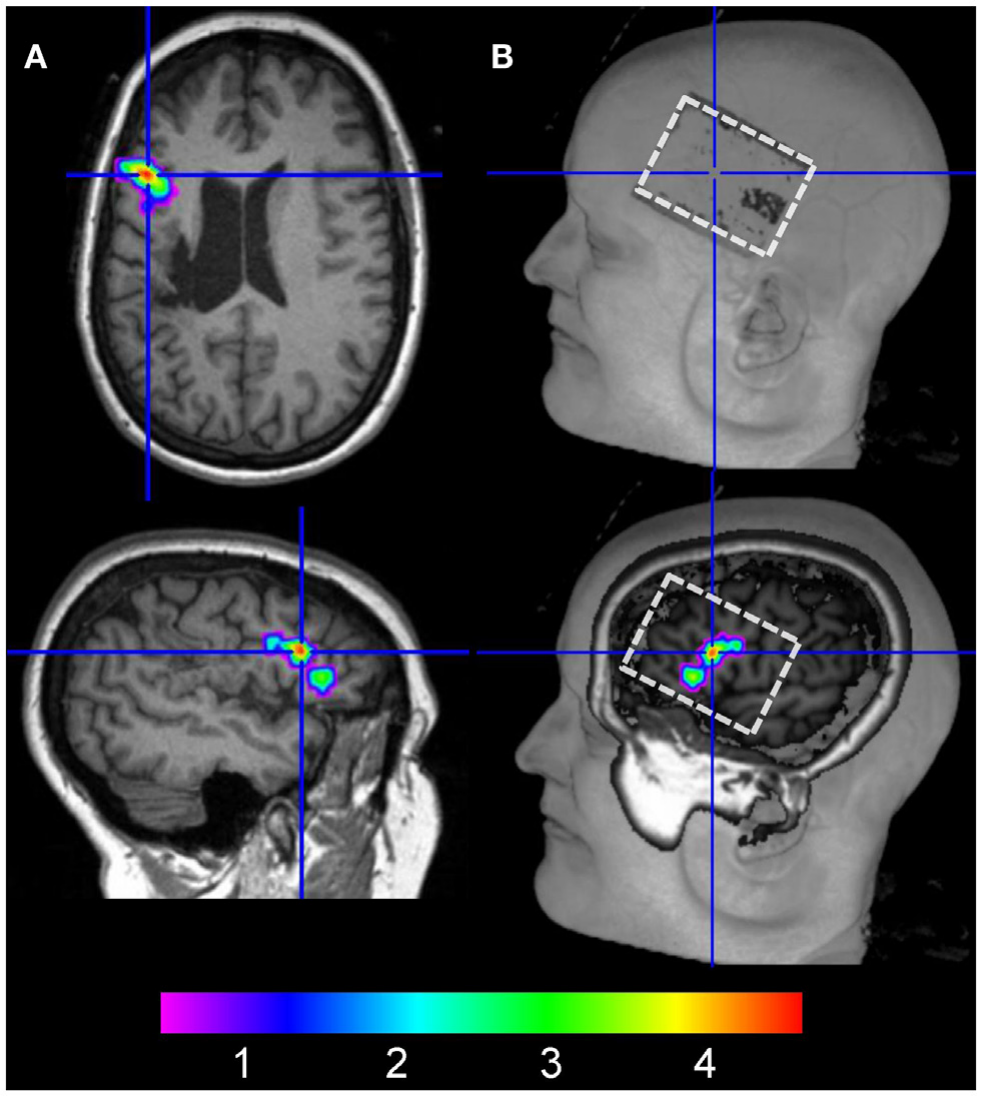
**(A)** The left column illustrates the location of peak activity in the left inferior frontal gyrus during the baseline fMRI session in native space (crosshair on axial and sagittal slices). **(B)** shows the location of the electrode on the scalp on the co-registered structural image of the patient acquired during anodal-tDCS (upper right image, white box). The lower right panel shows a more medial view of the same image with peak activity during the baseline scan overlaid.

To assure that the active electrode was located above the peak activity cluster identified during the baseline scan, the T1-weighted image acquired during the third fMRI session (anodal-tDCS) was co-registered with the respective T1-image acquired during the baseline scan. Peak activity obtained during the baseline scan (Figure 3A) was then overlaid on the T1-weighted image to allow for visual inspection of the baseline activity pattern relative to the electrode position during anodal-tDCS. This revealed that the electrode was placed correctly over the targeted area (Figure 3B).

### Performance during the cross-over phase

Picture stimuli included in the cross-over imaging phase were matched for response latency during the baseline sessions and only pictures with the fastest response latency were included to assure stable imaging results. Therefore, no differences were expected between the stimulation conditions. This was confirmed by comparing the number of correct responses (#sham-tDCS: 75; anodal-tDCS: 76) and response latency (mean ± SD milliseconds sham-tDCS: 1280 ± 223, anodal-tDCS: 1300 ± 237, *p* > 0.512), which were comparable between the two stimulation conditions.

### Task-related functional activity during the cross-over phase

**Figure 4:**
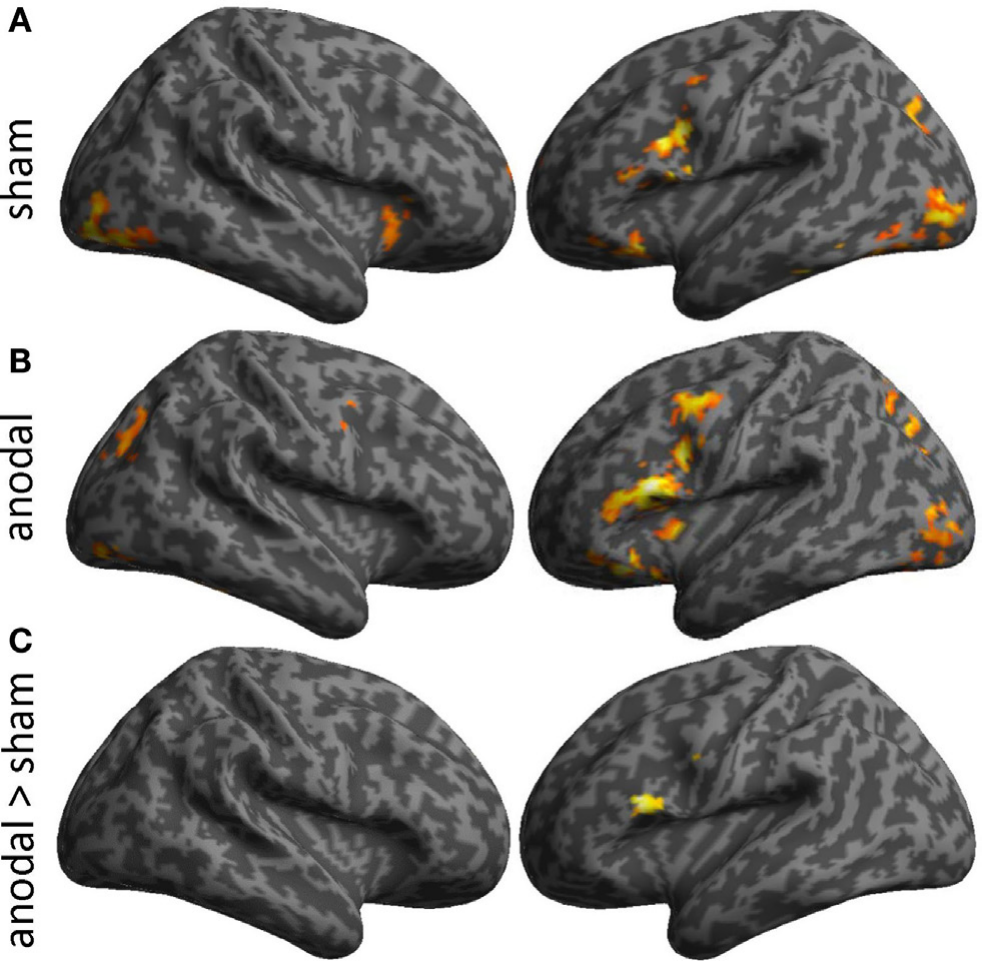
**Illustrates activity patterns associated with the two fMRI sessions as surface rendering overlaid on an inflated standard brain: (A) Sham-tDCS session, (B) Anodal-tDCS**. **(C)** The lower panel illustrates the location of increased task-related activity during anodal- vs. sham-tDCS. Baseline activity patterns and differences between sessions are thresholded at *p* < 0.001 at the voxel level and only clusters surviving a family wise-error corrected cluster threshold of *p* < 0.05 are shown.

Task-related activity associated with correct naming attempts during both sessions is detailed in Table 1 and Figures 4A,B. Overall, during both sessions, a highly consistent pattern of functional activity was found that was most pronounced in left frontal and occipital regions. Right frontal activity was only observed during sham-tDCS, whereas activity in the bilateral striatum was only active during anodal-tDCS. However, the direct comparison of both stimulation conditions revealed selectively increased activity in the left IFG (Brodmann areas 45/9, cluster extent *k* = 151 voxels, *Z* = 4.84, *x*/*y*/*z* = −51/20/19) during anodal-tDCS as compared to sham-tDCS (Figure 4C). No increased activity was detected for the inverse contrast.

**Table 1:** **Details of activity patterns associated with correct naming attempts **>** baseline for the two stimulation conditions (both thresholded *p* **<** 0.001 at voxel level, clusters surviving a family wise-error corrected cluster threshold of *p* **<** 0.05 are reported)**.

Session	Hemi	Structure	BA	*k*	*Z*	*x*	*y*	*z*
Sham-tDCS	L	Putamen		402	6.43	−16	2	5
	L	Inferior frontal gyrus	45	1671	6.12	−53	18	18
		Precentral gyrus	6		5.65	−38	3	24
		Inferior frontal gyrus	45		5.42	−46	22	8
	L	Inferior frontal gyrus	47	285	6.05	−36	23	−13
		Inferior frontal gyrus	47		4.52	−26	17	−14
		Middle frontal gyrus	11		3.84	−42	34	−13
	R	Caudate nucleus		729	5.94	14	4	9
		Putamen			5.11	16	6	2
	L	Superior frontal gyrus	6	911	4.92	−12	15	64
		Cingulate gyrus	32		4.90	−4	16	42
		Medial frontal gyrus	6		4.85	−4	27	35
	L	Precuneus	19	187	4.77	−30	−68	29
		Superior occipital gyrus	19		4.19	−30	−72	22
	R	Middle occipital gyrus	19/18	377	4.69	−40	−83	12
	R	Precuneus	19/7	319	4.09	26	−72	31
Anodal-tDCS	R	Inferior/middle occipital gyrus	18/37	632	6.41	44	−76	−10
	L	Inferior frontal gyrus	9	472	5.92	−40	5	24
			45		5.49	−53	26	12
	L	Middle occipital gyrus	18/37	720	5.65	−36	−87	1
	L	Precentral gyrus	44	154	5.44	−48	8	9
	L	Inferior frontal gyrus	47	221	5.38	−36	21	−14
		Middle frontal gyrus	11		4.25	−44	36	−19
		Inferior frontal gyrus	47		4.11	−40	28	−18
	R	Superior/medial frontal gyrus	10	252	5.11	10	61	23
	L	Medial frontal gyrus	10	316	4.66	−8	63	12
	R	Insula	13	249	4.51	32	23	1
		Inferior frontal gyrus	47		4.21	34	21	−6
	R	Middle frontal gyrus	46	238	4.47	59	28	23
		Superior frontal gyrus	9		4.19	34	48	33

*Hemi, hemisphere; BA, Brodmann’s area; *k* = cluster extent; *Z* = peak voxels in significant clusters, *x*/*y*/*z* coordinates in Talairach space*.

## Discussion

This proof-of-principle study demonstrated that it is feasible to precisely target an individually determined stimulation site during fMRI in post-stroke aphasia and to assess the neural underpinnings of acute tDCS effects. Importantly, picture stimuli used during the cross-over phase were closely matched for linguistic criteria and individual naming latency which assured that the (neural) stimulation effect was independent of performance. Indeed, during both stimulation sessions, the patient performed close to ceiling levels and task-related functional activity patterns were highly consistent. However, the direct comparison of anodal- and sham-tDCS revealed a selective increase of activity at the stimulation site (i.e., the left IFG). This suggests that anodal-tDCS modulated neural functioning during the overt picture naming tasks even in the absence of treatment or behavioral effects. Given that anodal-tDCS is thought to decrease the threshold required for neural depolarization at the stimulation site (Stagg and Nitsche, 2011), the most likely “mechanistic” explanation for enhanced IFG activity is that tDCS facilitated neural firing of this task-relevant region, resulting in a net increase of activity. Thus, the present study provides evidence for one possible mechanism that may have interacted with treatment approaches employed in previous clinical trials (Baker et al., 2010; Fridriksson et al., 2011). Importantly, functional imaging studies have highlighted the importance of increased task-related activity in perilesional regions after successful therapy (Meinzer et al., 2008; Fridriksson, 2010). Thus, facilitation of these areas by means of anodal-tDCS may be a viable way to enhance recovery even in chronic patients. Another important outcome of this study was that intrascanner stimulation allowed verification of correct electrode positioning over the target area, which was not possible in previous clinical trials.

Although the results of this study are based on a single patient with a small posterior lesion and well-recovered language functions, this approach may in the future allow identification of distinct patterns of neural responses associated with tDCS effects in larger cohorts of patients. Naturally, different lesions result in different patterns of functional reorganization, i.e., different stimulation sites will be targeted in other patients and the results will most likely vary from the current report. However, such studies may ultimately yield information about the variability of stimulation effects on brain functions in post-stroke aphasia and their relation to individual lesion patterns, functional reorganization, and language status. Given the high variability of stimulation effects in previous clinical tDCS trials (De Aguiar et al., 2015), a better understanding of the mechanisms by which tDCS impacts on brain function is highly desirable and also a pre-requisite for more targeted stimulation protocols in future clinical trials that combine tDCS with specific treatment.

Importantly, our set-up did not address the impact of the stimulation on performance. However, it allows identifying brain regions that are affected by a given montage on an individual basis to identify regions, which may potentially interact with speech therapy effects. Nonetheless, future studies could use this technique to assess the neural underpinnings associated with tDCS-induced performance improvements as previously demonstrated in healthy individuals (Holland et al., 2011; Meinzer et al., 2012a, 2013). Moreover, interactions with different tasks can be explored and optimized designs could be employed. For example, in the present study, anodal-tDCS was administered during the third imaging session. Given that repeated fMRI sessions are typically associated with reduced activity (Meltzer et al., 2009), it is unlikely that increased activity at the stimulation site during this session is explained by a simple order effect. However, additional scanning sessions with sham-tDCS would have been required to examine this. In addition, previous studies that employed the same intrascanner design have demonstrated that anodal-tDCS can also induce changes in functional connectivity (Meinzer et al., 2013) that cannot be captured by the univariate data analysis approach employed the present study. Unfortunately, the block design with equally spaced picture naming onsets prevented advanced task-related functional connectivity analyses. It also needs to be acknowledged that acute stimulation effects observed in the current study are mediated by different mechanisms than those underlying repeated administration of tDCS during treatment. Specifically, acute tDCS effects are mediated by modulation of neural resting membrane potentials which outlast the end of the stimulation only for short periods of time, whereas modulation of post-synaptic connections have been suggested to mediate long-lasting effects associated with repeated stimulation sessions (Stagg and Nitsche, 2011). However, despite the transient nature of acute stimulation effects, such studies allow us to identify which brain regions are modulated by the stimulation to allow for more evidence driven stimulation approaches in future treatment studies, even in individual patients.

## Conclusion

This study demonstrated that it is feasible to target an individualized stimulation site in post-stroke aphasia during simultaneous fMRI to assess the underlying neural signatures of tDCS-action in post-stroke aphasia. Although the results of this study are based on a single subject, this intrascanner stimulation approach provides researchers with a flexible tool to identify the neural mechanisms underlying stimulation effects in larger cohorts of patients, with the ultimate goal to optimize stimulation parameters in clinical trials that combine tDCS with different types of treatment.

## Conflict of Interest Statement

The authors declare that the research was conducted in the absence of any commercial or financial relationships that could be construed as a potential conflict of interest.
